# In Vitro Gastrointestinal Digestion of *Grifola frondosa* Polysaccharides and Their Enhancement of GABA Production via Gut Microbiota Modulation

**DOI:** 10.3390/nu17213332

**Published:** 2025-10-23

**Authors:** Qingchi Wang, Yuhang Luo, Huabo Zhu, Xiaoyang Liu, Mingyuan Xue, Guiling Yang, Yue Chen, Shiguo Chen, Zhengshun Wen

**Affiliations:** 1 Laboratory of Food Processing and Engineering, Department of Food Science and Nutrition, College of Biosystems Engineering and Food Science, Zhejiang University, Hangzhou 310058, China; 2 Xianghu Laboratory, Hangzhou 311231, China; 3 State Key Laboratory of Marine Food Processing & Safety Control, College of Food Science and Engineering, Ocean University of China, Qingdao 266400, China; 4 School of Food and Pharmacy, Zhejiang Ocean University, Zhoushan 316022, China; 5 Horticulture Research Institute, Zhejiang Academy of Agricultural Sciences, Hangzhou 310021, China

**Keywords:** *Grifola frondosa* polysaccharides, gut microbiota, digestion, fermentation, γ-aminobutyric acid

## Abstract

**Background**: The water-soluble *Grifola frondosa* polysaccharides (GFPs) are the primary bioactive component of the edible and medicinal fungus *Grifola frondosa*. However, the digestive behavior of GFPs in the human gastrointestinal (GI) tract and their subsequent interaction with gut microbiota (GM) to exert health effects remain unclear. **Methods**: In this study, GFPs were extracted based on a traditional hot water decoction. An in vitro simulated GI digestion model and a human fecal microbiota fermentation model were established to systematically investigate the digestive stability of GFPs, GM modulation, and metabolite changes. **Results:** Results showed that GFPs remained structurally stable during in vitro oral, gastric, and small intestinal digestion, allowing them to reach the colon intact for microbial fermentation. During colonic fermentation, GFPs were efficiently degraded by GM, and significantly increased the relative abundance of beneficial bacteria such as *Akkermansia*, *Bacteroides*, *Parabacteroides*, and *Lactobacillus* while reducing the abundance of pathogenic *Escherichia-Shigella*. Meanwhile, GFPs enriched metabolites beneficial for intestinal health, among which γ-aminobutyric acid (GABA) was the most significantly upregulated. Single-strain fermentation confirmed that *Lactobacillus* (*L. plantarum*) was the core GABA-producing genus. **Conclusions:** This study highlights the potential of GFPs as prebiotics for GM modulation, expands the understanding of the health-promoting effects of fungal polysaccharides, and provides a theoretical basis for the development of GFP-based functional foods.

## 1. Introduction

As a traditional Chinese fungus with both edible and medicinal values, *Grifola frondosa* has a history of consumption and application spanning nearly 2000 years. Its traditional and mainstream consumption method is water decoction, which allows it to serve not only as a daily food ingredient, but also to exert regulatory effects on the human body. Research has confirmed that *Grifola frondosa* possesses abundant health-promoting properties, and its polysaccharides are recognized as its core bioactive components. *Grifola frondosa* polysaccharides (GFPs) have been verified to exhibit multiple beneficial biological activities including the inhibition of tumor cell proliferation, the regulation of immune function, and the reduction in blood glucose levels [1,2]. From the perspective of food functionality, GFPs act as both a dietary fiber and a special nutrient, and they can exert biological activities regulating the compositional balance of gut microbiota (GM) [1,3]. For instance, in a diabetic mouse model, GFPs significantly increased the relative abundance of *Turicibacter* and *lactobacilli*, which facilitated the restoration of disrupted gut homeostasis [4]. In another study focusing on oxazolone-induced ulcerative colitis in mice, GFPs showed a clear ameliorative effect. This effect was achieved by modulating the GM structure, inhibiting the production of inflammatory mediators and promoting gut barrier repair [5]. Most existing studies have primarily focused on verifying the biological activities of GFPs and exploring their mechanisms. However, since GFPs must undergo gastrointestinal (GI) digestion before interacting with GM, systematic research on their specific digestive and metabolic processes remains lacking.

In fact, the GM plays an irreplaceable role in maintaining human health, and its functions are involved in multiple physiological processes. For example, GM mediates the absorption and utilization of nutrients from food, participates in the precise regulation of immune responses, contributes to vitamin synthesis, and modulates energy metabolism [6,7]. For dietary polysaccharides that are difficult for humans to digest directly, the role of GM is even more crucial. GM can degrade and utilize polysaccharides such as β-glucan, pectin, inulin, and fucoidan by encoding a variety of carbohydrate-active enzymes (CAZymes) [8,9]. This degradation process not only provides essential energy for the growth and proliferation of GM [9], but also generates metabolites that act as key signaling molecules for endocrine activities [10,11]. Thus, the GI tract and its resident microbiota serve as the hub connecting polysaccharide intake and the exertion of their efficacy.

In research exploring the interaction between polysaccharides and GM, in vitro digestion and fermentation models are crucial tools that effectively meet the research requirements. These models can precisely simulate the physiological conditions of the human oral, gastric, and intestinal digestive systems. Such simulations enable the dynamic analysis of changes in polysaccharide composition, degradation patterns, and interactions with GM, thereby facilitating the assessment of polysaccharide bioavailability [12]. Compared with in vivo experiments, in vitro models also possess significant advantages including a short experimental cycle, high efficiency, strong controllability of experimental conditions, and high repeatability and stability of results [12,13]. These advantages allow in vitro models to eliminate interference from the complex in vivo physiological environment, efficiently and clearly clarify the specific mechanisms by which prebiotic polysaccharides exert health-promoting effects in the human body [14,15], and provide reliable technical support for investigating the digestive and metabolic processes of polysaccharides.

In this study, the traditional water decoction method was employed for *Grifola frondosa* to align with its actual consumption practices. On this basis, ethanol precipitation was used to enrich the polysaccharide components from the aqueous extract, followed by deproteinization treatment. Subsequently, in vitro simulated GI digestion and GM fermentation systems were established. By monitoring indicators such as polysaccharide degradation rate and molecular weight changes, the digestive properties of water-soluble GFP were determined. Meanwhile, high-throughput sequencing and metabolomic analysis were employed to investigate the effects of GFP on human GM composition, the relative abundance of dominant flora, and metabolite changes. This study aimed to clarify the key mechanism underlying the transition of water-soluble GFPs from digestion to functional exertion, thereby laying a theoretical foundation for the application of GFPs in the development of functional foods.

## 2. Materials and Methods

### 2.1. Materials

The fruiting bodies of *Grifola frondosa* were gathered in Qingyuan, Zhejiang, China (geographic coordinates: 27°50′ N–28°29′ N, 119°17′ E–119°58′ E). Dextran standards were ordered from the National Institutes for Food and Drug Control (Beijing, China). D-mannose (Man), L-rhamnose (Rha), D-glucuronic acid (GlcA), D-galactosamine hydrochloride (GalN), D-glucosamine hydrochloride (GlcN), D-galacturonic acid (GalA), D-glucose (Glc), D-galactose (Gal), D-arabinose (Ara), L-fucose (Fuc), D-xylose (Xyl), acetic acid, propionic acid, and butyric acid were ordered from Macklin (Shanghai, China). Pepsin, α-amylase, gastric lipase, bile salts, pancreatin, deuterium oxide, and potassium bromide were ordered from Sigma-Aldrich (St. Louis, MO, USA). The Coomassie Brilliant Blue Assay Kit was ordered from Solarbio (Beijing, China). Sulfuric acid, phenol, and ether were ordered from Sinoharm Chemical Reagent Co., Ltd. (Shanghai, China).

### 2.2. Preparation of GFPs

The fresh fruiting bodies of *Grifola frondosa* were fully dried, ground using a high-speed grinder, and sieved through a 100-mesh sieve. The resulting powder was suspended in ultrapure water at a solid-to-liquid ratio of 1:40 (*w*/*v*) and stirred uniformly. The mixed solution was extracted at 100 °C for 2 h. After extraction, the mixture was filtered and centrifuged to remove precipitates. The supernatant was concentrated using rotary evaporation and then precipitated with anhydrous ethanol (1:4, *v*/*v*) at 4 °C for 12 h. The precipitate was collected, redissolved in deionized water, and treated with Sevag reagent (chloroform: N-butanol = 4:1, *v*/*v*) to remove free protein. The supernatant was dialyzed with a cut-off molecular weight of 3.5 kDa to remove small molecular impurities. Briefly, the deproteinized solution was transferred into the pre-activated dialysis membrane (MWCO 3.5 kDa, Solarbio, Beijing, China). The membrane was sealed tightly to prevent leakage and immersed in a large volume of ultrapure water in a glass beaker. The dialysis system was placed on a magnetic stirrer (80 rpm) at 4 °C to ensure the uniform diffusion of small molecules. The ultrapure water was replaced every 6 h for a total dialysis duration of 48 h. After dialysis, the retentate in the membrane was collected for subsequent concentration and freeze-drying to obtain the *Grifola frondosa* polysaccharides (GFPs).

### 2.3. Analysis of Physicochemical Properties of GFPs

#### 2.3.1. Basic Composition Determination

The total sugar content of GFPs was determined via the phenol-sulfuric acid method, as described in Reference [16]. Briefly, glucose (Glc) standard solutions (100 μg/mL) of 0, 0.1, 0.2, 0.3, 0.4, and 0.5 mL were separately pipetted into tubes, and each tube was made up to a final volume of 0.5 mL with ultrapure water. Subsequently, 0.5 mL of 5% (*w*/*v*) of phenol solution was added to each tube, followed by thorough mixing. A total of 2.5 mL of concentrated sulfuric acid was rapidly added to each tube, and the mixture was vortexed immediately to ensure homogeneity. The tubes were then heated at 100 °C for 10 min. After cooling to room temperature, the absorbance (OD values) of each solution was measured at 490 nm using a SpectraMax iD5 spectrophotometer (Molecular Devices, LLC, Sunnyvale, CA, USA). A standard curve was constructed with the sugar content (μg/mL) as the x-axis and the corresponding OD value as the y-axis. For sample analysis, GFP samples were prepared into 100 μg/mL solutions and treated identically to the Glc standards. The OD value of each sample was recorded at 490 nm, and the total sugar content of the GFPs was calculated by interpolating the sample OD value into the standard curve (*n* = 3).

The Bradford method was carried out to determine the protein content [17]. The tubes were added with different volumes (0, 0.01, 0.02, 0.04, 0.06, 0.08, 0.1 mL) of 0.5 mg/mL bovine serum albumin (BSA) standard protein solution. Each tube was then supplemented to 0.1 mL with ultrapure water. Then, 5.0 mL of Coomassie Brilliant Blue G-250 reagent was added to each tube, and the tubes were immediately mixed. Using a SpectraMax iD5 spectrophotometer, the absorbance values of the solutions in each tube were determined at 595 nm. A standard curve was plotted using the concentration of the BSA solution as the x-axis and the corresponding absorbance value as the y-axis. The sample was diluted to an appropriate range so that the measured values fell within the linear range of the standard curve. G-250 reagent was added to the diluted sample solution and mixed thoroughly. According to the measured absorbance value, the concentration of the protein to be measured could be calculated by comparing it with the standard curve (*n* = 3). The protein content analysis of the extracted GFPs was primarily aimed at evaluating the purity of the polysaccharide fraction; more importantly, it was to eliminate the potential interference of residual proteins on subsequent gut microbiota (GM) fermentation assays and downstream analytical procedures, ensuring that the experimental results accurately reflected the biological effects of GFPs.

#### 2.3.2. Molecular Weight Distribution Detection

The molecular weight (Mw) distribution of GFPs was determined by the gel permeation chromatography (GPC) method with a Shimadzu LC 2050C 3D series system (Shimadzu, Kyoto, Japan) equipped with a TSKgel G3000PW_XL_ column (Tosoh, Tokyo, Japan). GFP and dextran standards were filtered through a filter with a 0.22 μm membrane. A total of 0.1 M Na_2_SO_4_ solution served as the mobile phase, flowing at a rate of 0.5 mL/min. The Mw for each GFP peak was calculated based on a standard curve, plotted with the logarithmic values of the dextran molecular weight (2700, 5250, 9750, 13,050, 36,800, 64,650, 135,350, and 300,600 Da) on the y-axis and corresponding retention times on the x-axis.

#### 2.3.3. Monosaccharide Composition Analysis

The monosaccharides contained in the GFPs were measured using the 1-phenyl-3-methyl-5-pyrazolone (PMP) pre-column derivatization method [18]. GFP solution was mixed with 4 mol/L trifluoroacetic acid (TFA) (1: 1, *v*/*v*) in an ampoule bottle and heated at 115 °C for 4 h. The degradation fluid was concentrated repeatedly using a vacuum concentrator (Martin Christ Gefriertrocknungsanlagen GmbH, Osterode am Harz, Lower Saxony, Germany) to remove excess TFA. The sample was redissolved in water, mixed with 0.3 mol/L sodium hydroxide, and 0.5 mol/L PMP methanol solution (1:1:1.2, *v*/*v*/*v*). This mixture was incubated at 70 °C for 1 h, and adjusted the pH 7.0 by 0.3 mol/L hydrochloric acid. Excess PMP was removed by extraction with chloroform and centrifugation (12,000× *g*, 10 min, room temperature). The upper aqueous phase (containing PMP-derivatized monosaccharides) was filtered through a 0.22 μm nylon membrane (MilliporeSigma, Billerica, MA, USA) to remove impurities. This filtered aqueous phase served as the final solvent for injection. The mixed monosaccharide standards (D-mannose, L-rhamnose, D-glucuronic acid, D-galactosamine hydrochloride, D-glucosamine hydrochloride, D-galacturonic acid, D-glucose, D-galactose, D-arabinose, L-fucose, D-xylose) with gradient concentrations (5, 10, 20, 40, 80, 160 μmol/L) were derived directly with PMP, and other operations were consistent with the sample.

A Shimadzu LC 2050C 3D series system with a PDA detector (Shimadzu, Kyoto, Japan) was used to separate and quantify the PMP-derivatized monosaccharides with the following detailed parameters: the chromatographic separation was performed on a ZORBAX SB-AQ C18 column (4.6 mm × 250 mm, 5 μm; Agilent Technologies, Santa Clara, CA, USA) maintained at 30 °C, with a mobile phase consisting of 0.1 mol/L phosphate-buffered saline (PBS, pH 6.8) and acetonitrile at a volume ratio of 83:17 (*v*/*v*, %). The mobile phase was delivered at a constant flow rate of 0.8 mL/min, and the injection volume of the filtered PMP-derivatized sample was 10 μL. Detection was carried out using a PDA detector set at a wavelength of 245 nm to quantify the PMP-derivatized monosaccharides.

A standard curve was constructed with monosaccharide concentration (μmol/L) on the x-axis and the peak area of the PMP derivative on the y-axis to generate the linear equation y = ax + b (R^2^ ≥ 0.999). Target monosaccharides in the samples were identified by their retention times relative to the PMP-derivatized standards. Each peak area was inserted into the corresponding equation to obtain the molar concentration, which was multiplied by the injection volume to yield the molar amount. The monosaccharide present in the smallest amount was set as the reference (1.00), and the ratios of all other monosaccharides to this reference were calculated to give the molar composition of the GFPs.

#### 2.3.4. Fourier Transform Infrared Spectrum Analysis

GFP powder was completely dried in a vacuum environment at 50 °C for 24 h. The dried powder was scanned within the range of 4000 to 400 cm^−1^ using a Nicolet iS50 Fourier transform infrared (FT-IR) spectrometer (Thermo Fisher Scientific, Waltham, MA, USA) [19].

#### 2.3.5. Congo Red Assay

The Congo red assay was conducted to investigate the structural properties of GFPs, with a specific focus on the detection of the triple-helical structure of β-glucan [20]. Briefly, a 10 mg/mL GFPs solution was prepared using ultrapure water, and a 0.3 mol/L Congo red standard solution was prepared separately. Subsequently, 0.9 mL of the GFP solution, 0.5 mL of the Congo red solution, and 1.6 mL of ultrapure water were mixed in a tube, followed by incubation at room temperature for 10 min. After incubation, the mixed solution was transferred to a quartz colorimetric tube, and the maximum absorption wavelength (λ_max_) of the Congo red–GFP complex was determined using a spectrophotometer at wavelength range from 400 to 650 nm.

For the pH-dependent binding analysis, 1 mol/L NaOH solution was added to the Congo red–GFP mixture according to Tables S1 and S2. The λ_max_ value was recorded for each point, and the binding affinity between the GFPs and Congo red was evaluated based on the change in λ_max_ relative to the blank control. A significant red shift in λ_max_ was considered a positive result, indicating the presence of β-glucan in GFPs. All detections were performed in triplicate.

### 2.4. In Vitro Simulated Digestion

The in vitro simulation of GFP digestion in the gastrointestinal (GI) tract was performed with reference to the method established by André Brodkorb et al. [21], which enables the realistic reproduction of the full process of human GI digestion.

#### 2.4.1. Oral Digestion

Simulated salivary fluid (SSF) was made up of calcium chloride dihydrate (1.5 mmol/L) and α-amylase (75 U/mL), followed by adjusting the pH to 7.0. GFP solution (6 mg/mL) was mixed with the SSF in a 1:1 ratio by weight and digested in a shaking incubator at 37 °C for 2 min. Aliquots of the digestion solution were collected at time points of 0 min and 2 min, and the enzyme was deactivated by boiling for 5 min (*n* = 3).

#### 2.4.2. Gastric Digestion

Simulated gastric fluid (SGF) was prepared by adding calcium chloride dihydrate (0.15 mmol/L), pepsin (2000 U/mL), and gastric lipase (60 U/mL) to pre-warmed distilled water at 37 °C, followed by adjusting the pH to 3.0. The previously prepared SSF was then mixed with the SGF in a 1:1 ratio by volume and incubated in a shaking incubator at 37 °C for 2 h. The digestion solution of each sample was collected at 0, 1, and 2 h, and the enzyme was deactivated by boiling for 5 min (*n* = 3).

#### 2.4.3. Small Intestine Digestion

Simulated small intestinal fluid (SIF) was prepared by pre-warming distilled water to 37 °C, then adding calcium chloride dihydrate (0.6 mmol/L) and trypsin (100 U/mL), and adjusting the pH to 7.0. The previously prepared SGF was mixed with SIF in a 1:1 ratio. Subsequently, bile salts (10 mmol/L) were added to the solution, which was then digested at 37 °C for 2 h. The digestion solution of each sample was collected at 0, 1, and 2 h, and the enzyme was deactivated by boiling for 5 min (*n* = 3).

To test whether GFPs are digested after each stage of digestion, GFPs were precipitated from the digestive fluid by adding absolute ethanol at a volume ratio of 1:4 (digest:ethanol) for the determination of monosaccharide composition.

### 2.5. In Vitro Simulated Fermentation of GFPs

The fermentation of GFPs was conducted using a method improved by Wang et al. [22]. Fecal samples were collected from 12 healthy adult donors (aged 21–28 years) with no history of gastrointestinal disease, antibiotic or probiotic use, or prebiotic supplementation in the 3 months prior to sampling. All donors followed a regular mixed diet and were non-smokers and non-drinkers. All participants were informed about the content of the study and signed informed consent.

Fresh feces were carefully transferred into 50 mL sterile tubes within 3 min of defecation to ensure immediate sealing and weighed. Equal amounts of fecal samples from 12 donors were pooled and homogenized with sterile PBS to create a 10% (*w*/*v*) suspension. Residues in the suspension were gently removed by filtration through a 0.4 mm mesh. Subsequently, 1.0 mL of the filtrate was inoculated into 50 mL of the medium to initiate the fermentation process. Fermentation was performed in an Electrotek AW 500TG anerobic chamber (Electrotek Manufacturing, Shipley, West Yorkshire, UK) with an atmosphere of 80% N_2_, 10% CO_2_, and 10% H_2_. Redox condition was automatically monitored and controlled by the built-in system, and all media and consumables were pre-equilibrated inside the chamber.

The medium containing GFPs (6 mg/mL) was designated as the treatment group (GFPs), the inulin-supplemented medium was designated the positive control group (INL), and the medium without GFPs/INL was designated the blank control group (BLK). Samples from each group were collected at 0, 6, 12, 24, and 48 h. Each sample was centrifuged at 8000× *g* for 20 min, and the supernatant and pellet were stored separately at −80 °C (*n* = 3).

### 2.6. Dynamic Monitoring of GFPs During Digestion and Fermentation

The determination of reducing sugars in each sample was carried out using the dinitrosalicylic acid method, as described in the literature [23]. The procedures for evaluating the total sugar content, molecular weight (Mw) distribution, and monosaccharide composition of fermentation broth were conducted in accordance with previously described methods. The pH was measured using a pH meter (Mettler-Toledo International Inc., Zurich, Switzerland).

### 2.7. Determination of SCFAs in Fermentation Broth

Short-chain fatty acids (SCFAs) were extracted using an improved protocol based on a previously reported method [24]. Briefly, 500 μL of fermentation broth was acidified with 20 μL of 10% sulfuric acid, followed by extraction with 800 μL of ether. The mixture was subjected to high-speed centrifugation (4 °C, 18,000× *g*, 20 min). The supernatant was then analyzed using a GCMS-TQ8050 NX gas chromatography-mass spectrometry system (Shimadzu, Kyoto, Japan), equipped with an SH-Rtx-WAX gas chromatography column (Restek, Bellefonte, PA, USA; 30 m × 0.25 mm × 0.25 μm). The analytical parameters were as follows: helium served as the carrier gas; the column temperature was maintained at 60 °C for 3 min, ramped to 150 °C at a rate of 8 °C/min and maintained for 2 min, then increased to 220 °C at a rate of 40 °C/min and maintained for 4 min. SCFAs were quantified using GC-MS with 2-ethylbutyric acid (1.0 mmol/L) as the internal standard. Calibration curves for acetic, propionic, and butyric acids were prepared at concentrations of 0.05–5.0 mmol/L. All calibration curves exhibited linearity with R^2^ > 0.99. The LOD and LOQ were determined based on signal-to-noise ratios of 3 and 10, respectively, ranging from 0.005 to 0.015 mmol/L (LOD) and 0.015 to 0.05 mmol/L (LOQ). Each group was tested in triplicate (*n* = 3), and the results are expressed as the mean ± standard error of the mean (SEM).

### 2.8. 16S rDNA Sequencing of Gut Microbiota

DNA extraction was conducted using an E.Z.N.A. Stool DNA Kit (Catalog No. D4015, Omega Bio-tek, Norcross, GA, USA). The extracted total DNA was stored at −80 °C for subsequent analysis. As shown in Table S3, the V3–V4 hypervariable region of the bacterial 16S rRNA gene was amplified using the universal primer pair 341F (5′-CCTACGGGNGGCWGCAG-3′) and 805R (5′-GACTACHVGGGTATCTAATCC-3′) (synthesized by Tsingke Biotechnology, Beijing, China). The 5′ ends of the primers were labeled with Illumina adapter sequences to enable subsequent sequencing on the Illumina NovaSeq platform. Conventional PCR (not real-time PCR) was used for amplification. The PCR reaction system (25 μL) contained 12.5 μL of 2×Taq Plus Master Mix (Vazyme Biotech, Nanjing, China), 1 μL of each primer (10 μmol/L; synthesized by Tsingke Biotechnology, Ltd., Beijing, China), 2 μL of template DNA (50 ng/μL), and 8.5 μL of nuclease-free water. PCR conditions: initial denaturation at 95 °C for 5 min; 30 cycles of denaturation at 95 °C for 30 s, annealing at 60 °C for 30 s, extension at 72 °C for 45 s; final extension at 72 °C for 10 min. The PCR products were verified by 1.5% agarose gel electrophoresis, purified using a DNA Gel Extraction Kit (Axygen Scientific, Hangzhou, China), and quantified with a Qubit 4 Fluorometer (Thermo Fisher Scientific, Waltham, MA, USA) before sequencing. The amplified samples were analyzed on the Illumina NovaSeq platform (Illumina, San Diego, CA, USA) to obtain results for the GM composition. The OmicStudio cloud platform (https://www.omicstudio.cn, accessed on 12 September 2024) was used for the visualization of these sequencing results.

### 2.9. Non-Targeted Metabolites Analysis of Fermentation Fluid

The metabolite detection method referred to the previously reported protocol [25]. The fermented supernatant was mixed with a precooled organic solvent mixture (acetonitrile: methanol = 1:1, stored at −20 °C) in a 1:4 (*v*/*v*) ratio, vortexed for 40 s, and facilitated metabolite extraction at −20 °C for 90 min. The mixture was centrifuged at 15,000× *g* for 15 min at 4 °C, and the supernatant was dried. Sample was re-extracted in 0.2 mL of 80% acetonitrile, left undisturbed for 30 s, and then centrifuged again under the same conditions. The samples were filtered through a 0.22 μm membrane for subsequent analysis. Equal volumes of extracts from each group were pooled to create quality control (QC) samples. Metabolites from each group were chromatographically separated using an UltiMate 3000 UPLC system (Thermo Fisher Scientific, Inc., Waltham, MA, USA) with an ACQUITY UPLC T3 column (100 mm × 2.1 mm, 1.8 µm, Waters Corporation, Milford, MA, USA) for reversed-phase separation at 40 °C. Acetonitrile was used as the mobile phase with a flow rate of 0.3 mL/min, and samples were eluted under gradient conditions (0–2 min: 2% acetonitrile, 2–10 min: 2–98% acetonitrile, 10–12 min: 98% acetonitrile, 12–12.1 min: 98–2% acetonitrile, 12.1–15 min: 2% acetonitrile). Metabolites were detected using a Q-Exactive mass spectrometer (Thermo Fisher Scientific, Inc., Waltham, MA, USA) with the following parameters: electrospray ionization (ESI) source in both positive and negative ion modes; spray voltage: 3.5 kV (positive ion mode) and 3.2 kV (negative ion mode); capillary temperature: 320 °C; sheath gas flow rate: 40 arb; auxiliary gas flow rate: 10 arb; S-lens RF level: 50; full scan range: m/z 70–1050; resolution: 70,000 (full scan) and 17,500 (MS/MS scan); data-dependent acquisition (DDA) mode with dynamic exclusion (exclusion duration: 30 s). After every ten samples, a quality control (QC) sample was tested to ensure both the integrity of the acquisition process and the reliability of the LC-MS system (*n* = 3).

Metabolite information annotation was performed using the Kyoto Encyclopedia of Genes and Genomes (KEGG, https://www.genome.jp/kegg/, accessed on 9 October 2024) and Human Metabolome Database (HMDB, https://hmdb.ca/, accessed on 8 October 2024), with annotations considered valid if the mass deviation was less than 10 ppm between the observed and database values. Data analysis and visualization of the mass spectrometry results were carried out using R software (version 4.0). Calculation of the variable importance in projection (VIP) was performed using partial least squares discriminant analysis (PLS-DA) with the R package ropls (Version 1.24.0). Thresholds for selecting significantly differential metabolites were set as *p* < 0.05, VIP > 1, and |log_2_^FC^| > 1, and a volcano plot was generated based on these criteria. Visualization of the KEGG pathways was accomplished using Metaboanalyst 6.0 (https://www.metaboanalyst.ca/, accessed on 15 October 2024).

### 2.10. Single Bacterial Fermentation

*Parabacteroides distasonis, Bacteroides thetaiotaomicron, Akkermansia muciniphila,* and *Lactobacillus plantarum* (BNCC, Xinyang, Henan, China) were cultured in GFP-added basal medium [22,26] with the following main components: GFP, 6.0 mg/mL; yeast extract, 4.0 mg/mL; peptone, 3.5 mg/mL; tryptone, 3.0 mg/mL; dehydrated beef heart infusion, 3.0 mg/mL; dehydrated calf brain infusion, 3.0 mg/mL; NaCl, 4.0 mg/mL; KCl, 2.5 mg/mL; KH_2_PO_4_, 0.5 mg/mL; MgSO_4_, 0.5 mg/mL; CaCl_2_, 0.1 mg/mL; Tween 80, 1 mL/L; and trace elements solution, 0.2 mL/L. The trace elements solution contained FeSO_4_·7H_2_O, 0.2 mg/mL; ZnSO_4_·7H_2_O, 0.18 mg/mL; CoCl_2_·H_2_O, 0.10 mg/mL; CuSO_4_·5H_2_O, 0.01 mg/mL; and MnCl_2_·4H_2_O, 0.02 mg/mL. Specifically, for each of the four strains (*L. plantarum*, *B. thetaiotaomicron*, *P. distasonis*, *A. muciniphila*), six biological replicates (*n* = 6) were set up in the GFP-added basal medium to ensure data reliability. The GABA content, pH and optical density at 600 nm (OD_600_) were measured at 0, 6, 12, 24, 48, and 72 h, respectively. One-way analysis of variance (ANOVA) followed by Tukey’s post hoc test was used to compare differences in GABA production between strains at the same time point.

### 2.11. Determination of GABA in the Fermentation Broth by LC-MS/MS

Quantitative determination of GABA was carried out using a Waters triple quadrupole liquid chromatography-mass spectrometry instrument Xevo TQ−XS (ESI source, positive ion mode). The chromatographic column selected was a Waters ACQUITY UPLC BEH C18 column (2.1 mm × 150 mm, 1.7 μm). The column temperature was set at 35 °C, the injection volume was 1 μL, and the flow rate was 0.3 mL/min. A gradient elution was performed using 0.1% formic acid aqueous solution (phase A)−acetonitrile (phase B) as the mobile phase (0–3 min, 98% A; 3–6 min, 98% A→20% A; 6–8 min, 20% A; 8–10 min, 98% A). In mass spectrometry, the MRM mode was adopted to monitor the ion pairs. The quantitative transitions of GABA were m/z 104.1→87.1 (cone voltage 22 V, collision energy 8 eV), and the qualitative ion pair was m/z 104.1→69.2 (cone voltage 22 V, collision energy 15 eV). The source parameters were set as follows: capillary voltage 3.0 kV, source temperature 120 °C, desolvation gas temperature 350 °C, desolvation gas flow rate 800 L/h, and cone gas flow rate 50 L/h.

Take 200 μL of the supernatant of the fermentation broth, add 4-fold volume of cold acetonitrile, let it stand at 4 °C for 10 min, then centrifuge at 12,000 rpm and 4 °C for 15 min. Take the supernatant, add 20 μL of 1 mol/L sodium bicarbonate for neutralization, let it stand at 4 °C for 5 min, and then filter through a 0.22 μm membrane before injecting into the machine for detection (*n* = 3). For GABA quantification, use authentic GABA standard (purity ≥ 99%, Sigma-Aldrich, St. Louis, MO, USA) as the external standard: prepare a series of GABA standard solution concentrations (100, 500, 1000, 2000, 5000, 10,000 ng/mL) with 50% acetonitrile–water and analyze these standard solutions under the same LC-MS/MS conditions as the fermentation samples. Plot a standard curve with the GABA concentration as the abscissa and the peak area of GABA as the ordinate, and calculate the GABA concentration in the sample through MassLynx™ software (v4.2). GABA levels were expressed as mg/L, and the values were normalized to the fermentation broth volume to reflect the total metabolic output per unit volume.

### 2.12. Statistical Analysis

The Kruskal–Wallis and Wilcoxon tests were used as the basis for linear discriminant analysis (LEfSe), which was employed to assess the effect size. Statistical significance across multiple groups was assessed using one-way analysis of variance (ANOVA). After one-way ANOVA showed significant differences among groups (*p* < 0.05), Tukey’s post hoc test was used for pairwise comparisons to identify which specific groups differed with GraphPad Prism 10 (Boston, MA, USA). Results were expressed as the mean ± SEM and considered statistically significant when *p* < 0.05/*p* < 0.01.

## 3. Results

### 3.1. Prepared and Physicochemical Properties Analysis of GFPs

**Figure 1:**
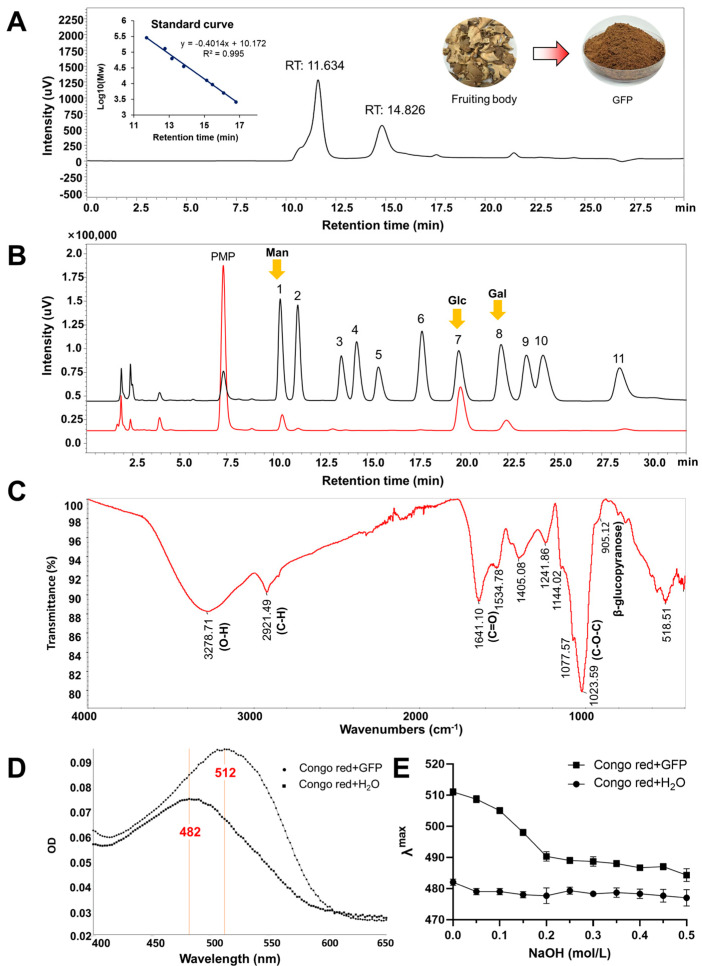
Analysis of the physicochemical properties of *Grifola frondosa* polysaccharides (GFPs). (**A**) Gel permeation chromatography (GPC) analysis was performed using a TSKgel G4000PWxl column (7.8 mm × 300 mm) with 0.1 mol/L Na_2_SO_4_ as the mobile phase (flow rate: 0.5 mL/min, column temperature: 35 °C), and detection via a refractive index detector (RID−20A, Shimadzu). (**B**) Monosaccharide composition was determined via HPLC (Shimadzu LC−2050) after 1−phenyl−3−methyl−5−pyrazolone (PMP) pre-column derivatization (70 °C reaction for 1 h, pH adjusted to 7.0 with 0.3 mol/L HCl) using a ZORBAX SB-AQ C18 column (4.6 mm × 250 mm, 5 μm) and mobile phase of 0.1 mol/L phosphate-buffered saline (pH 6.8):acetonitrile = 83:17 (*v*/*v*) (flow rate: 0.8 mL/min, detection wavelength: 245 nm). Mannose (Man), glucose (Glc) and galactose (Gal) marked by yellow arrows are the dominant monosaccharides in the GFPs. Peaks corresponded to the following monosaccharides: 1−Man, 2−GlcN, 3−Rha, 4−GlcA, 5−GalA, 6−GalN, 7−Glc, 8−Gal, 9−Xyl, 10−Ara, 11−Fuc. (**C**) Fourier transform infrared (FTIR) spectrum of the GFPs (scanning range: 4000–400 cm^−1^). (**D**) Maximum absorption wavelength (λ_max_) scanning profiles of the Congo red solution and GFP–Congo red complex. (**E**) Variation trend of λ_max_ for the Congo red solution and GFP–Congo red complex in the presence of different concentrations of NaOH (0.1–0.5 mol/L).

After hot water extraction, ethanol precipitation, and deproteinization, the final yield of *Grifola frondosa* polysaccharides (GFPs) were 6.4% relative to the dried fruiting bodies. The total sugar content, determined by the phenol-sulfuric acid method and expressed as glucose equivalents, was 75.40% ± 0.35%, while the protein content, measured via the Bradford method, was 17.01% ± 0.91%. Gel permeation chromatography (GPC) analysis revealed that the GFPs exhibited two distinct chromatographic peaks, with corresponding average molecular weights (Mw) of 317.8 kDa (accounting for 68%) and 16.6 kDa (accounting for 32%), respectively (Figure 1A). Monosaccharide composition analysis indicated that the GFPs were predominantly composed of mannose (Man), glucosamine (GlcN), glucose (Glc), galactose (Gal), and fucose (Fuc), with a molar ratio of 6.09:1.00:35.96:7.51:2.04 (Figure 1B). Among these, Man, Glc, and Gal were the dominant monosaccharides in the GFPs.

The functional group information of GFPs were identified using Fourier transform infrared (F-IR) spectroscopy. As shown in Figure 1C, the broad absorption peak at 3278.71 cm^−1^ corresponded to the O−H stretching vibration, while the sharp absorption peak at 2921.49 cm^−1^ was attributed to the C−H stretching vibration. Three observed peaks at 1144.02 cm^−1^, 1077.57 cm^−1^, and 1023.59 cm^−1^ indicated the stretching vibration of C−O−C (cyclic ether), suggesting that GFP is mainly composed of pyranose rings. The peak at 1641.10 cm^−1^ was tentatively assigned to the C=O stretching vibration of the acetylamino group (−NHCOCH_3_) in GlcN. Additionally, a weak absorption observed at 905.12 cm^−1^ indicated the C-H bending vibration associated with β-glycosidic linkages in GFPs.

β-Glucan is a key bioactive polysaccharide in mushrooms. When Congo red binds to β-glucan with a triple- helical structure, its conjugate system expands, resulting in a redshift of the maximum absorption wavelength (λ_max_). As presented in Figure 1D, the interaction between GFPs and Congo red caused a redshift of λ_max_ from 482 nm to 512 nm, indicating that GFPs possess a specific triple-helical structure. An alkaline environment can disrupt the binding interaction between GFPs and Congo red. When NaOH solutions of different concentrations (0.1–0.5 mol/L) were added, a blueshift of λ_max_ was observed for the GFP–Congo red complex (Figure 1E). These results confirm that GFPs contain the β-glucan fraction.

### 3.2. Characteristics of GFPs During In Vitro Simulated Digestion

**Figure 2:**
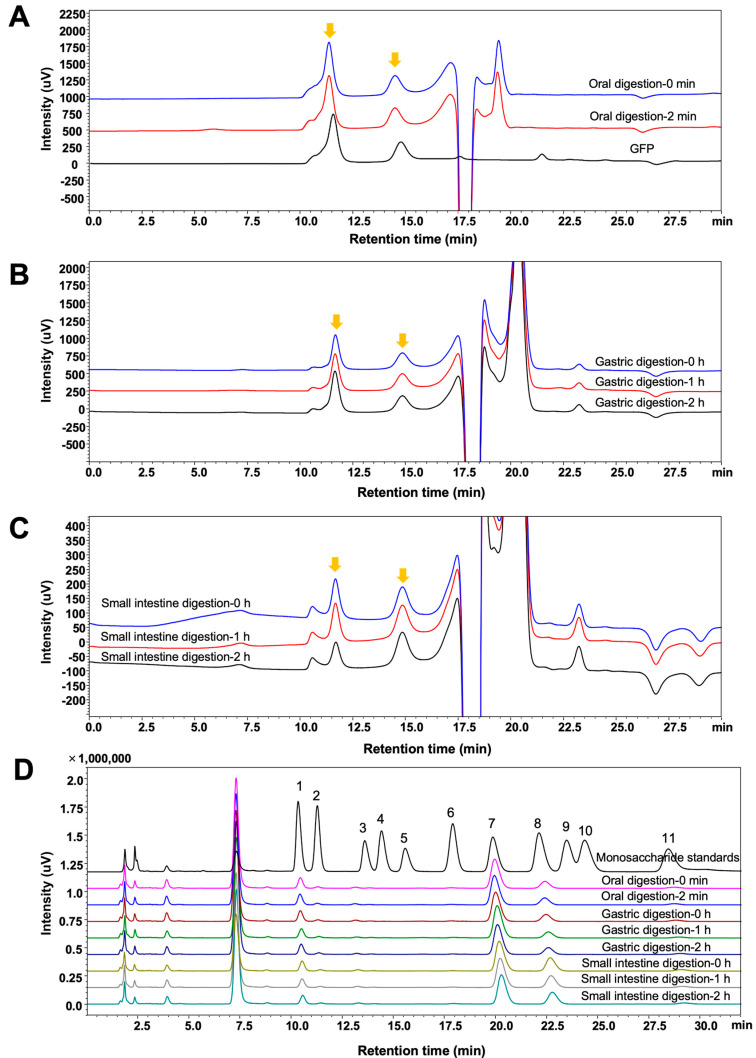
Changes in the molecular weight distribution and monosaccharide composition of *Grifola frondosa* polysaccharides (GFPs) during the in vitro simulated digestion. The molecular weight distribution of GFPs during simulated oral digestion (**A**), gastric digestion (**B**), and small intestinal digestion (**C**). The chromatographic peaks of GFPs are marked with yellow arrows. (**D**) Monosaccharide composition of GFPs across different simulated digestion stages (oral: 0 min, 2 min; gastric: 0 h, 1 h, 2 h; small intestinal: 0 h, 1 h, 2 h). Peaks corresponded to the following monosaccharides: 1−Man, 2−GlcN, 3−Rha, 4−GlcA, 5−GalA, 6−GalN, 7−Glc, 8−Gal, 9−Xyl, 10−Ara, 11−Fuc.

The digestion of GFPs in the oral cavity, stomach, and small intestine was simulated in vitro. During this process, the molecular weight distribution, monosaccharide composition, total sugar content, and reducing sugar content of GFPs were monitored. As shown in Figure 2A–C, the two peaks corresponding to the different molecular weight distributions of GFPs exhibited no significant changes at different time points in the simulated saliva, gastric juice, and small intestinal juice, indicating that the GFPs remained stable during the simulated digestion stage.

The monosaccharide composition of the GFPs was monitored across different digestion stages. To determine this composition, polysaccharides were precipitated from the digestive fluid by adding absolute ethanol at a volume ratio of 1:4 (digest:ethanol). As shown in Figure 2D, the peak intensities of the three main monosaccharides (Man, Glc, and Gal) in GFPs did not show significant changes at different time points, suggesting that the GFPs maintained structural integrity throughout the entire digestion process.

Changes in the total and reducing sugar contents of the digestive fluid are key indicators for assessing the digestibility of GFPs. As shown in Table 1, no significant fluctuations were observed in the total sugar or reducing sugar levels of GFPs during the simulated oral, gastric, and small intestinal digestion stages. These results confirm the stability of GFPs in the gastrointestinal (GI) tract. Specifically, GFPs resist hydrolysis by human genome-encoded enzymes in the upper GI tract [27], thereby reaching the colon and creating opportunities for interaction with the gut microbiota (GM).

### 3.3. Interaction Between GFPs and Gut Microbiota During In Vitro Fermentation

#### 3.3.1. Dynamic Changes During GFPs Fermentation

**Figure 3:**
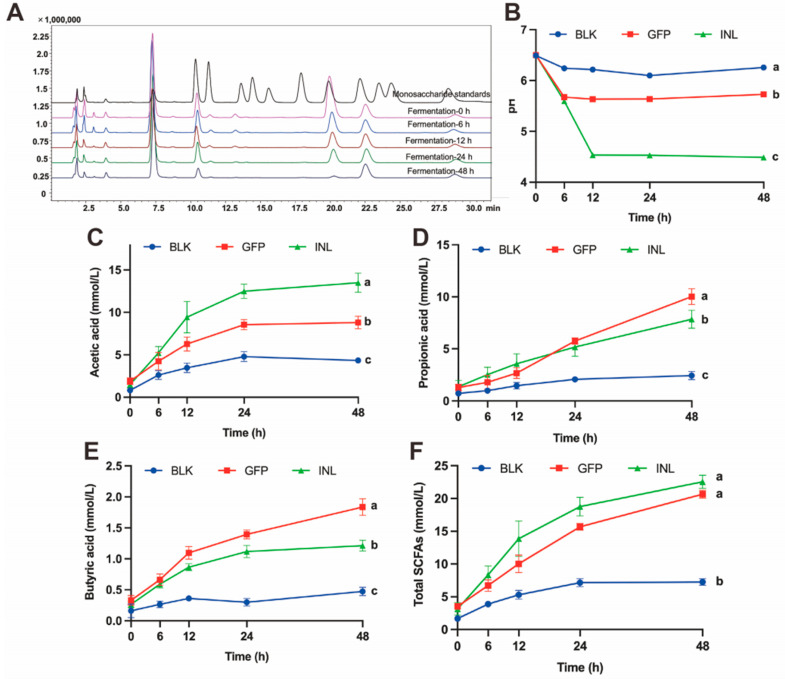
Dynamic changes in the fermentation broth properties during the in vitro fermentation of GFPs. (**A**) Changes in the monosaccharide composition of GFPs at different fermentation time points (0 h, 6 h, 12 h, 24 h, 48 h, peaks corresponded to the following monosaccharides: 1−Man, 2−GlcN, 3−Rha, 4−GlcA, 5−GalA, 6−GalN, 7−Glc, 8−Gal, 9−Xyl, 10−Ara, 11−Fuc). (**B**) Changes in the pH value of the fermentation broth across three groups [blank control (BLK), GFPs, positive control (INL, inulin-supplemented)] at different fermentation time points (0 h, 6 h, 12 h, 24 h, 48 h). pH was measured at 0, 6, 12, 24, 48, and 72 h using an SD20 pH meter (Mettler-Toledo, accuracy: ±0.01 pH unit), with 3 technical replicates per group. Changes in acetic acid (**C**), propionic acid (**D**), butyric acid (**E**), and total SCFA (**F**) content of the fermentation broth across the BLK, GFP, and INL groups at different fermentation time points (0 h, 6 h, 12 h, 24 h, 48 h). Short-chain fatty acids (SCFAs) were detected via gas chromatography-mass spectrometry (GC-MS, Thermo TRACE 1310−ISQ QD) using an SH-Rtx-WAX column (30 m × 0.25 mm × 0.25 μm). The letters a, b, c indicate significant differences, meaning values labeled with different letters are significantly distinct from each other.

Colonic fermentation of GFPs was simulated using mixed fecal samples from healthy adults. Concurrently, changes in the total sugar content, reducing sugar content, monosaccharide composition, pH, and short-chain fatty acid (SCFAs) levels of the fermentation broth were monitored. As fermentation progressed, both the total sugar and reducing sugar content of GFPs decreased progressively (Table 1). This decline indicates that the GM continuously degraded and utilized GFPs. Subsequently, we investigated which monosaccharides were preferentially utilized by the GM. As shown in Figure 3A, with the extension of fermentation time, the levels of Man, Glc, and GlcN in the GFPs decreased significantly, with Glc nearly completely depleted at 48 h. In contrast, the levels of Gal and Fuc remained relatively stable. This suggests that the GM exhibits selectivity in utilizing the monosaccharides within GFPs. Specifically, Glc is the most preferred, followed by Man and GlcN, while Gal and Fuc are the lowest priority.

The pH is a crucial environmental indicator of the microbial habitat. The GM lowers the pH by metabolizing substrates to produce small molecules (e.g., organic acids and SCFAs), thereby creating a more favorable growth environment for itself [28]. As shown in Figure 3B, the pH values of the positive control (INL, inulin-supplemented) group and the GFP group changed significantly during fermentation, dropping from an initial pH of 6.5 to 4.48 and 5.72 at 48 h, respectively. Conversely, the pH of the control (BLK, no GFPs/inulin) group remained relatively stable.

SCFAs play a vital role in maintaining a low intestinal pH and promoting the growth of beneficial bacteria [29,30]. Gas chromatography-mass spectrometry (GC-MS) was used to quantitatively analyze the SCFAs during fermentation. The results showed that in both the GFP group and INL group, the levels of the three main SCFAs (acetic acid, propionic acid, and butyric acid) increased over time. The acetic acid concentration was highest in the INL group, followed by the GFP group (Figure 3C). After 24 h of fermentation, the propionic acid concentration in the GFP group increased significantly, exceeding that in the INL group (Figure 3D). Throughout the entire fermentation process, the butyric acid content was highest in the GFP group (Figure 3E). The total SCFA concentration reflects the overall fermentative activity and provides an integrated measure of microbial metabolic response to substrate. The increased total SCFAs observed with GFP supplementation indicate enhanced carbohydrate fermentation by the GM (Figure 3F). These results suggest that GFPs, acting as a prebiotic, interact with the GM to modulate its growth and metabolism, ultimately promoting the production of SCFAs that are beneficial to host health.

#### 3.3.2. Regulatory Role of GFPs in Modulating Gut Microbiota

To further explore the impact of GFPs on GM, we assessed the changes in GM diversity and abundance via 16S rDNA high-throughput sequencing. A total of 1,069,214 and 1,141,761 bases were identified from the three experimental groups at 0 and 48 h, respectively. At both time points, 99.9% of the sequences ranged in length from 400 to 500 bp (base pairs) (Table S4). Quality filtering was applied at a minimum Phred score of Q30 (Table S5). The rarefaction curves of all groups plateaued at the end, and the rank abundance curves further illustrated differences in the microbial diversity and community structure among groups (Figure S1A–C). These results indicate that the sequencing depth and data volume were sufficient for subsequent analyses.

**Figure 4:**
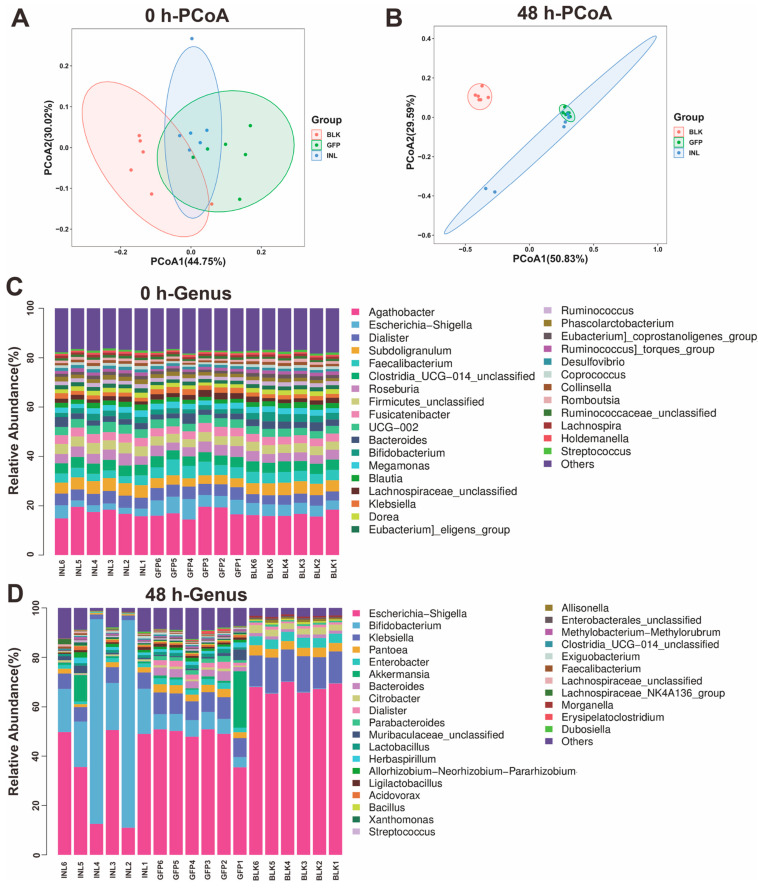
Analysis of gut microbiota (GM) diversity and community composition during the in vitro fermentation of GFPs. (**A**) β-Diversity analysis of GM composition via principal coordinate analysis (PCoA) based on UniFrac distances at 0 h. PCoA1 and PCoA2 explained 44.75% and 30.02% of the total variance, respectively. (**B**) β-Diversity analysis of GM composition via PCoA based on UniFrac distances at 48 h. PCoA1 and PCoA2 accounted for 50.83% and 29.59% of the total variance, respectively. (**C**) Relative abundance of GM at the genus level across three groups [blank control (BLK), GFPs, positive control (INL, inulin-supplemented)] at 0 h. (**D**) Relative abundance of GM at the genus level across the BLK, GFP, and INL groups at 48 h.

Principal coordinate analysis (PCoA) based on UniFrac distances revealed minimal intergroup differences at 0 h, with PCoA1 and PCoA2 explaining 44.75% and 30.02% of the total variance, respectively (Figure 4A). In contrast, at 48 h, PCoA1 and PCoA2 contributed for 50.83% and 29.59% of the variance, respectively (Figure 4B), indicating a significant shift in the microbial community structure. The increased distance between the GFP and BLK groups, along with the reduced distance between the GFP and INL groups, reflects distinct microbial community differentiation between GFPs and BLK, while GFPs and INL exhibited more similar community structures.

To further evaluate the influence of GFPs on human GM after 48 h of fermentation, α-diversity analysis was performed (Figure S1D,E). The GFP group exhibited significantly higher Chao1 and Shannon indices than the BLK group (*p* < 0.05), indicating greater species richness and diversity. This supports the hypothesis that GFPs promote a more diverse GM. Furthermore, the Shannon index of the GFP group was significantly higher than that of the INL group, demonstrating that GFPs enhance GM diversity more effectively than inulin.

We also investigated the GFP-induced changes in GM abundance during fermentation. At the onset of fermentation (0 h), the GM composition was similar across all groups, with five dominant genera: *Agathobacter*, *Subdoligranulum*, *Dialister*, *Faecalibacterium*, and *Escherichia-Shigella* (Figure 4C). After 48 h of fermentation, however, both the GFP and INL groups showed reduced relative abundances of *Escherichia-Shigella* and *Klebsiella* compared with the BLK group (Figure 4D and Figure S2). Additionally, the GFP group exhibited a significant increase in the relative abundance of *Akkermansia*, *Bacteroides*, *Lactobacillus*, *Parabacteroides*, and *Dialister* compared with the other two groups (Figure S2).

**Figure 5:**
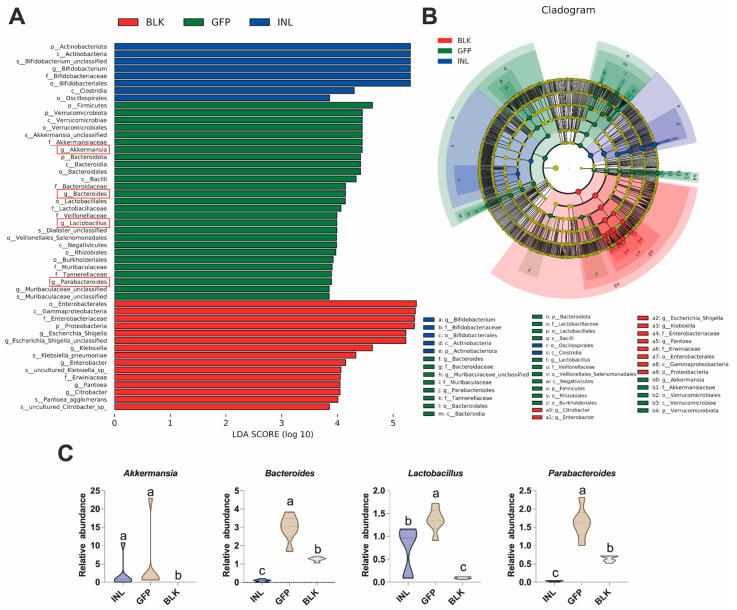
Key gut microbiota taxa regulated by GFPs after 48 h of in vitro fermentation. (**A**) Linear discriminant analysis (LDA) score histogram of differentially abundant GM taxa (LDA score > 3.5). The four key bacterial genera in the GFP group were highlighted with red rectangles. (**B**) Cladogram of LEfSe analysis showing taxonomically distinct GM taxa (LDA score > 3.5) across the three groups. (**C**) Box plots illustrating the relative abundance of four key beneficial genera (*Akkermansia, Bacteroides, Lactobacillus*, and *Parabacteroides*) identified through LEfSe analysis in the BLK, GFP, and INL groups. Different lowercase letters (a, b, c) indicate statistically significant differences between groups (*p* < 0.05).

To identify the dominant GM taxa in each group, we used linear discriminant analysis effect size (LEfSe) to detect taxonomically distinct taxa that differed significantly between groups. Here, we focused on taxa with a linear discriminant analysis (LDA) score > 3.5 (Figure 5A,B). The relative abundance of *Proteobacteria* (particularly the *Escherichia-Shigella, Klebsiella,* and *Enterobacter*) was significantly increased in the BLK group (Figure S3). In contrast, compared with the BLK and INL groups, the GFP group showed a significant enrichment of *Akkermansia*, *Bacteroides*, *Lactobacillus*, and *Parabacteroides* (Figure 5C), which is consistent with the results of the relative abundance analysis. These four genera are widely recognized as probiotics in the human intestine [31,32,33]. These findings suggest that GFPs can modulate the structure and composition of GM by increasing the relative abundance of specific beneficial bacteria.

#### 3.3.3. Effects of GFP on Metabolite Production

The gut harbors trillions of microorganisms that are crucial for breaking down complex carbohydrates, and their metabolites serve as a bridge between the microbiota and the host [34]. To investigate the regulatory effects of GFPs on GM-derived metabolites during in vitro fermentation, we employed an untargeted metabolomics approach in both positive and negative ion modes. A total of 16,603 metabolites were detected via liquid chromatography-mass spectrometry (LC-MS).

**Figure 6:**
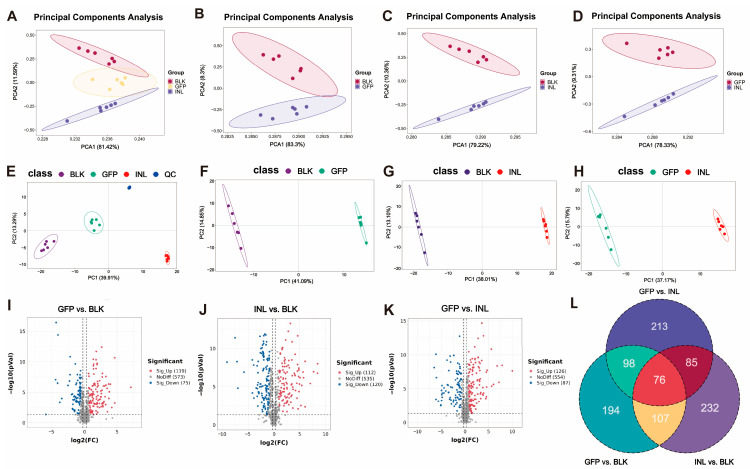
Effects of GFPs on gut microbiota metabolites during in vitro fermentation. (**A**) Principal component analysis (PCA) score plot showing the separation of the three groups (GFPs, INL, and BLK). (**B**–**D**) Pairwise PCA score plots: (**B**) GFP vs. BLK groups, (**C**) INL vs. BLK groups, (**D**) GFP vs. INL groups. PCA was performed using R software (v4.0.3) with the autoscaling of data (normalization to unit variance), and quality control (QC) samples were included to assess the system stability (RSD of QC samples < 15%). (**E**–**H**) Partial least squares discriminant analysis (PLS-DA) results: (**E**) PLS-DA score plot with quality control (QC) samples; pairwise PLS-DA score plots for (**F**) GFP vs. BLK groups, (**G**) INL vs. BLK groups, (**H**) GFP vs. INL groups. PLS-DA was conducted with 7-fold cross-validation and the permutation test (100 permutations, R^2^Y > 0.5, Q^2^Y < 0 to avoid overfitting) using the ropls package in R. The volcano plots showed the differential metabolites in (**I**) GFP vs. BLK groups, (**J**) INL vs. BLK groups, and (**K**) GFP vs. INL groups, with upregulation in red and downregulation in blue. (**L**) Venn diagram showing the distribution of unique significantly differential metabolites in the GFP, INL, and BLK groups (screening criteria: *p* < 0.05, VIP > 1, |log_2_^FC^| > 1).

Principal component analysis (PCA) was used to assess metabolite diversity. The results showed clear separation among the three experimental groups (Figure 6A). Pairwise PCA analyses further revealed distinct separation of the GFP group from the BLK and INL groups (Figure 6B–D). Subsequently, partial least squares discriminant analysis (PLS-DA) was applied to evaluate the metabolic differences between groups (Figure 6E–H). The PLS-DA score plot showed distinct clustering of the compared groups. Furthermore, permutation tests indicated that all models had R^2^ > 0.5 and negative Q^2^ values (Figure S4), indicating that none of the models were overfitted and that they could reliably capture the metabolic differences between groups.

Volcano plots were used to visually represent metabolites with significant differences between groups (Figure 6I–K). Using the screening criteria of *p* < 0.05, variable importance in projection (VIP) > 1, and |log_2_^FC^| > 1, we identified 194,232, and 213 unique significantly differential metabolites in the GFP, INL, and BLK groups, respectively (Figure 6L).

**Figure 7:**
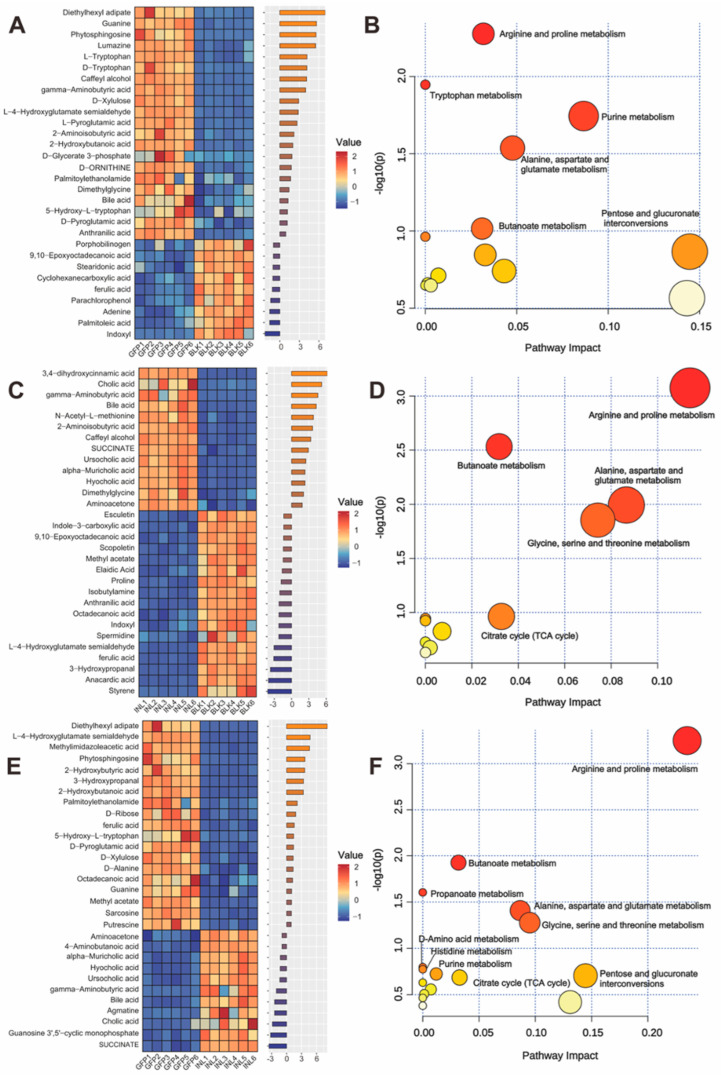
Comparative analysis of significantly differential metabolite profiles induced by GFPs. (**A**) Heatmap and bubble plot of the top 30 differential metabolites between the GFP and BLK groups. (**B**) Enriched metabolic pathways for the top 30 metabolites (GFPs vs. BLK) based on KEGG. (**C**) Heatmap and bubble plot of the top 30 metabolites between the BLK and INL groups. (**D**) Enriched pathways for the top 30 metabolites (INL vs. BLK) based on KEGG. (**E**) Heatmap and bubble plot of the top 30 metabolites between the GFP and INL groups. (**F**) Enriched pathways for the top 30 metabolites (GFP vs. INL) based on KEGG. Heatmaps use red for higher and blue for lower relative abundance. Pathway plots use darker colors to indicate a greater number of enriched pathways.

Enrichment analysis was performed on the top 30 metabolites from each comparison based on their *p*-value ranks. As shown in Figure 7A, compared with the BLK group, the GFP group mainly upregulated 2-hydroxybutanoic acid, γ-aminobutyric acid (GABA), and L-pyroglutamic acid and significantly downregulated ferulic acid, 9,10-epoxyoctadecanoic acid, and cyclohexanecarboxylic acid. The metabolites influenced by GFPs were significantly enriched in Kyoto Encyclopedia of Genes and Genomes (KEGG) pathways, particularly those involved in arginine and proline metabolism, tryptophan metabolism, and purine metabolism (Figure 7B). Compared with the BLK group, inulin significantly upregulated ursocholic acid, hyocholic acid, and α-muricholic acid and significantly downregulated ferulic acid, anthranilic acid, and L-4-hydroxyglutamate semialdehyde (Figure 7C). As a positive control, the INL group showed significant enrichment of affected metabolites in pathways such as arginine and proline metabolism, butanoate metabolism, and the TCA cycle (Figure 7D). Furthermore, compared with the INL group, the GFPs group significantly upregulated L-4-hydroxyglutamate semialdehyde, 2-hydroxybutanoic acid, and sarcosine while significantly downregulating hyocholic acid and α-muricholic acid (Figure 7E). The pathways that exhibited the greatest enrichment between the two groups were arginine and proline metabolism, butanoate metabolism, and propanoate metabolism (Figure 7F).

#### 3.3.4. GFPs Enhance GABA Production by Upregulating Beneficial Gut Microbiota

**Figure 8:**
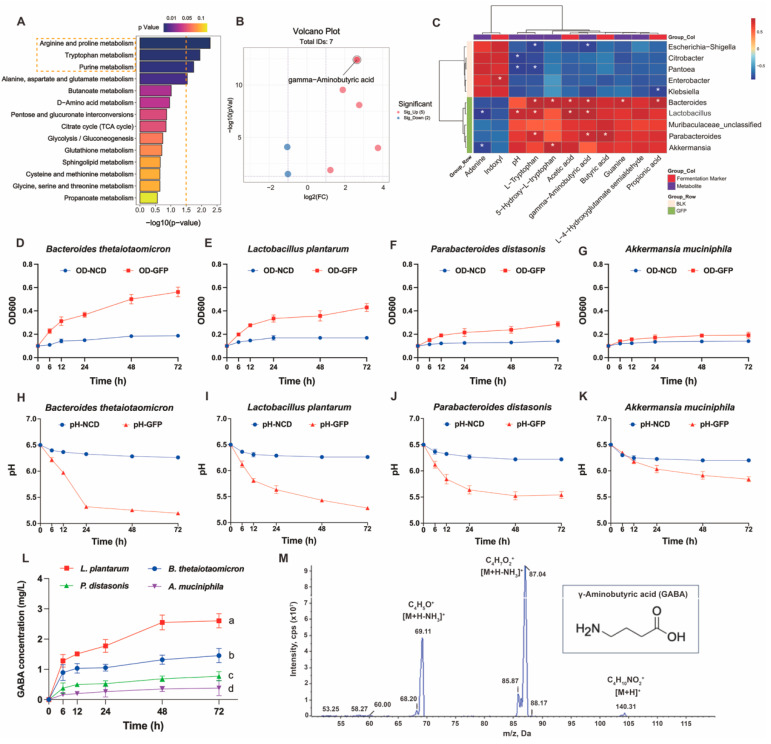
Regulatory effects of GFPs on gut microbiota (GM) –derived γ-aminobutyric acid (GABA) production. (**A**) Top enriched Kyoto Encyclopedia of Genes and Genomes (KEGG) pathways in the comparison of GFP vs. BLK groups (ranked by *p*-value). The top three KEGG metabolic pathways with the lowest *p*-values were highlighted with yellow rectangles. (**B**) Volcano plot of differential metabolites in the top three enriched KEGG pathways (*p* < 0.05, |log_2_^FC^| > 1). (**C**) Spearman correlation analysis between key bacterial genera, fermentation markers, and differential metabolites in the GFP and BLK groups. Red squares represent positive correlations, and blue squares represent negative correlations; * *p* < 0.05 indicates significant correlations. (**D**–**G**) OD_600_ values of *Bacteroides thetaiotaomicron*, *Lactobacillus plantarum*, *Parabacteroides distasonis*, and *Akkermansia muciniphila* during fermentation in GFP-supplemented medium. (**H**–**K**) pH changes of fermentation broths containing *B. thetaiotaomicron*, *L. plantarum*, *P. distasonis*, and *A. muciniphila*. (**L**) GABA concentration in fermentation broths of the four strains over time. (**M**) Multiple reaction monitoring (MRM) mass spectrometric signal profile of the GABA standard (transitions: *m*/*z* 104→69.2, *m*/*z* 104→87.1). The letters a, b, c, d indicate significant differences, meaning values labeled with different letters are significantly distinct from each other (*p* < 0.05).

To further identify the beneficial gut microbiota and their metabolites affected by GFPs, we first ranked the KEGG-enriched differential metabolic pathways (GFPs vs. BLK) by *p*-value. The results showed that arginine and proline metabolism, tryptophan metabolism, and purine metabolism were the top three differential pathways modulated by GFPs (Figure 8A). Using the thresholds of *p* < 0.05 and |log_2_^FC^| > 1, we plotted a volcano plot for the seven differential metabolites enriched in these pathways (Figure 8B, Table S6). Among these metabolites, γ-aminobutyric acid (GABA) was the most significant differential one. GABA is a well-recognized amino acid with multiple biological activities. We further conducted Spearman correlation analysis on ten key bacterial taxa, seven key metabolites, and four fermentation markers identified in the BLK and GFPs groups. As shown in Figure 8C, *Bacteroides*, *Lactobacillus*, and *Parabacteroides* exhibited a significant positive correlation with GABA, while *Akkermansia* showed a positive but non-significant correlation. Based on microbiome profiling and correlation analysis, we selected four representative commercial strains (*Bacteroides thetaiotaomicron, Lactobacillus plantarum, Parabacteroides distasonis,* and *Akkermansia muciniphila*) to verify whether GFPs could enhance microbial GABA production [33,35,36,37].

Single-strain fermentation was conducted for 72 h with GFPs as the sole carbon source, and the optical density (OD) value, pH value, and GABA content of the fermentation broth were monitored. As shown in Figure 8D–G, the OD_600_ values of *B. thetaiotaomicron, L. plantarum,* and *P. distasonis* increased gradually over time, indicating the continuous growth and proliferation of these three strains under the experimental conditions. In contrast, the OD_600_ value of *A. muciniphila* remained relatively stable throughout the process, with no significant proliferation or decline.

Changes in pH are closely associated with bacterial metabolic activity. As shown in Figure 8H–K, the pH values of the fermentation broths for *B. thetaiotaomicron, L. plantarum, P. distasonis,* and *A. muciniphila* all showed a downward trend over time. This is because these strains produced SCFAs during growth and metabolism, leading to a decrease in environmental pH, which also indirectly reflects their relatively active metabolic activities. As shown in Figure 8L, different strains exhibited varying GABA-producing capacities. The GABA content of *L. plantarum* increased significantly over time with a large amplitude of increase. The GABA contents of *B. thetaiotaomicron* and *P. distasonis* also increased to a certain extent, but their increase rates and final contents were lower than those of *L. plantarum*. In contrast, the GABA content of *A. muciniphila* showed little change, remaining at a relatively low level. Notably, lactic acid bacteria have been previously shown to produce GABA, which aligns with the observed GABA-synthesizing capacity of *L. plantarum* in our study [38].

In conclusion, our study shows that GFPs not only upregulate beneficial gut bacteria and bioactive metabolites, but also have a key role in enhancing GABA production, which is mainly mediated by increasing the abundance of GABA-producing *Lactobacillus*.

## 4. Discussion

The gut microbiota (GM) plays various essential roles in the human body and is often regarded as a “critical organ” [39]. The composition of the GM is highly dynamic and subject to modulation by multiple factors, among which dietary intake is a major determinant. Most dietary polysaccharides escape digestion and absorption by the host and rely on the metabolic capacity of the GM for utilization. Through fermentation, the microbiota converts these indigestible polysaccharides into a spectrum of metabolites that not only maintain microbial community homeostasis, but also confer benefits to host physiology [40,41]. This study employed an in vitro model to investigate the digestive properties of water-soluble *Grifola frondosa* polysaccharides (GFPs) and their interactions with GM, providing further evidence for the beneficial effects of GFPs for human health.

GFPs as complex carbohydrates cannot be directly digested by the upper human digestive tract and thus reach the colon, where they undergo specific microbial fermentation. Dynamic monitoring of monosaccharide composition during fermentation revealed that glucose (Glc) showed the most pronounced decline, indicating that it was preferentially utilized by the GM as a primary energy source. This observation is consistent with the metabolic characteristics of dominant genera such as *Bacteroides*, which secrete β-glucanases and preferentially cleave glycosidic bonds of glucose residues [42]. The simultaneous microbial degradation and utilization of GFPs resulted in the production of SCFAs, which altered the colonic pH [27]. Studies have shown that acetate, propionate, and butyrate are the three major SCFAs in the gut, accounting for approximately 90–95% of total SCFAs [43,44]. Acetate has been associated with improved glucose and lipid metabolism in the host, which may help prevent obesity [45]. Propionate is considered an effective substrate for gluconeogenesis in the liver and intestine [46]. Butyrate serves as the principal energy source for colonic epithelial cells and has been shown to not only reduce the incidence of ulcerative colitis, but also suppress the expansion of inflammation-associated microbial populations [44,47]. In this study, the butyrate concentration in the GFP group consistently exceeded that of the blank control (BLK) and the inulin positive control (INL) groups, and the propionate level also surpassed that in the INL group after 24 h of fermentation. This distinctive pattern of SCFA modulation highlights a core mechanism by which GFPs exert intestinal protective and metabolic regulatory effects, underscoring their unique advantages over conventional prebiotics.

To identify GM taxa that specifically responded to GFPs, we performed 16S rDNA sequencing on the fermentation broth. *Escherichia-Shigella*, a known pathogen associated with intestinal inflammation, showed a significantly higher relative abundance in the BLK group than in the GFP and INL groups. In contrast, GFPs reduced the relative abundance of *Escherichia-Shigella*. As common inhabitants of the human gut, *Escherichia-Shigella* can disrupt intestinal homeostasis when overgrown, leading to conditions such as diarrhea and enteritis [48,49]. The inhibitory effect of GFPs on this genus may be attributed to the SCFAs generated during fermentation, which lower the intestinal pH and create an acidic environment unfavorable for pathogenic growth [50,51]. Conversely, the relative abundances of *Akkermansia*, *Bacteroides,* and *Lactobacillus* were significantly higher in the GFP group than in the other two groups. *Akkermansia* is well-recognized for its beneficial role in regulating the intestinal barrier, alleviating intestinal inflammation, and addressing metabolic dysfunction associated with fatty liver disease [52]. An increase in its relative abundance is often regarded as a signal that the host is activating gut protective mechanisms [36]. Furthermore, *Bacteroides* is a major component of the GM. In addition to serving as “providers” that supply energy sources to the host and neighboring microorganisms, *Bacteroides* confers numerous health benefits by modulating the host’s metabolic networks [53]. As a probiotic genus with a long history of use, *Lactobacillus* has been widely applied in food fermentation and human health interventions. Moreover, *Lactobacillus* has been shown to enhance the antioxidant, lipid-lowering, and hypoglycemic activities of polysaccharides through structural modification [54]. These results support previous reports that fungal polysaccharides can inhibit the growth of intestinal pathogens while promoting the proliferation of beneficial gut bacteria [55].

The microbiota–metabolite axis represents the core mechanism by which the GM exerts profound effects on the host [56]. In this study, by integrating untargeted metabolomics, single-strain fermentation, and targeted validation, we comprehensively identified the characteristic metabolites influenced by GFPs, among which the marked enrichment of GABA proved particularly critical. GABA is a bioactive compound naturally present in the human body and various microorganisms, widely recognized for its anti-inflammatory and antihypertensive effects [57]. Additionally, foods rich in GABA are gaining popularity in the food industry [58]. However, the human body has a relatively limited capacity to synthesize GABA, with most of it produced by the GM through various metabolic pathways [59]. *Lactobacillus*, a “star” bacterial genus in the human gut, has been extensively documented to synthesize GABA via complex metabolic pathways involving glutamic acid decarboxylase (GAD) [60]. Notably, pH is one of the most critical environmental factors governing GABA production, as its biosynthetic pathway is tightly linked to pH [38]. This aligns with our observation that *Lactobacillus* cultured in GFP-containing medium exhibited lower pH values accompanied by elevated GABA concentrations. Moreover, *Lactobacilli* strains serve as a major platform for the commercial-scale production of GABA. For instance, in a study by Kook et al., *Lactobacillus sakei* expressing the GAD from *Lactobacillus plantarum* enabled the scale-up of GABA production from 5 L to 300 L [61]. Beyond the intrinsic activity of GAD, the viability of *Lactobacilli* also exerts a positive influence on GABA biosynthesis [62]. Interestingly, *L. plantarum* cultured in GFP-containing medium exhibited a markedly higher OD_600_ value than that in the control group. This effect may be attributed to the diverse glycoside hydrolases in *L. plantarum*, which enable the degradation and metabolism of GFPs, thereby facilitating GABA production [63]. A limitation of this study is the absence of a negative control addressing the potential contribution of proteins or simple sugars. Although GFPs showed structural stability during digestion and fermentation patterns were consistent with polysaccharide utilization, the influence of co-extracted components cannot be fully excluded. Future studies will incorporate enzymatically hydrolyzed GFP controls or matched sugar/protein controls to dissect the relative contributions of intact polysaccharides and their degradation products.

The interplay between GFPs, GM, and GABA holds substantial translational value rooted in traditional wisdom and modern scientific validation. GFPs, as the core bioactive component of *Grifola frondosa* (a medicinal food with 2000-year history in East Asia), align with traditional applications targeting “spleen nourishment” and “qi regulation”, whose effects have now been linked to GM modulation. Our findings that GFPs enrich GABA-producing strains and elevate GABA levels bridge traditional gut–nervous system insights with the modern gut–brain axis theory. This connection is reinforced by mushroom polysaccharides’ well-documented prebiotic activity, as they selectively promote beneficial bacteria and enhance short-chain fatty acid production to improve intestinal health and intervene in non-communicable diseases [64,65].

Commercially, this axis addresses booming demands in functional food markets. The global GABA food market is projected to grow at 8.5% compound annual growth rate (CAGR) by 2030, driven by sleep disorders, stress management needs, and youth health concerns. Unlike direct GABA addition, GFPs modulate GM to sustain GABA production, fitting “clean-label” trends while avoiding reliance on synthetic additives. Notably, GFPs’ synergy with probiotics (enhancing *Lactobacillus* growth and GABA secretion) enables synbiotic development, addressing probiotic survival challenges in the gastrointestinal tract, which is a critical industrial bottleneck. Such synbiotics could target diverse populations from adults seeking stress relief (via GABA-mediated cortical regulation) to children for growth support [66].

Although future translation requires clinical validation (e.g., human trials on sleep or gut discomfort) and technological refinement, current evidence positions the GFP–GM–GABA axis as a promising framework for functional food and supplement innovation.

## 5. Conclusions

In this study, *Grifola frondosa* polysaccharides (GFPs) extracted via the traditional decoction method exhibited stability in the upper gastrointestinal tract. Upon reaching the colon, GFPs are fermented by gut microbiota, leading to the production of beneficial metabolites. Meanwhile, GFPs increased the abundance of beneficial bacteria including *Bacteroides*, *Lactobacillus*, *Akkermansia*, and *Parabacteroides* and significantly enhanced γ-aminobutyric acid (GABA) production, primarily by enriching *Lactobacillus*. The increased GABA production may be one of the main reasons why GFPs exert their health effects. Collectively, this study provides a research basis for the in-depth development of GFPs in functional foods.

## Figures and Tables

**Table 1:** Changes in the total sugar and reducing sugar content of GFPs during in vitro digestion and fermentation. Different lowercase letters (a–e) indicate statistically significant differences between groups (*p* < 0.05).

	Time	Total Sugars (mg/mL)	Reducing Sugars (mg/mL)
Oral digestion	0 min	0.6565 ± 0.0059 ^a^	0.1198 ± 0.0121 ^a^
	2 min	0.6685 ± 0.0216 ^a^	0.1201 ± 0.0191 ^a^
Gastric digestion	0 h	0.3759 ± 0.0035 ^a^	0.1192 ± 0.0192 ^a^
	1 h	0.3711 ± 0.0129 ^a^	0.1181 ± 0.0301 ^a^
	2 h	0.3754 ± 0.0078 ^a^	0.1212 ± 0.0212 ^a^
Small intestinal digestion	0 h	0.2476 ± 0.0021 ^a^	0.1109 ± 0.0141 ^a^
	1 h	0.2427 ± 0.0078 ^a^	0.1094 ± 0.0231 ^a^
	2 h	0.2453 ± 0.0047 ^a^	0.1091 ± 0.0133 ^a^
Fecal fermentation	0 h	0.5594 ± 0.0336 ^a^	1.8533 ± 0.1704 ^a^
	6 h	0.1779 ± 0.0126 ^b^	0.4533 ± 0.0850 ^b^
	12 h	0.0751 ± 0.0211 ^c^	0.2330 ± 0.0497 ^c^
	24 h	0.0351 ± 0.0352 ^d^	0.0147 ± 0.0047 ^d^
	48 h	0.0115 ± 0.0137 ^e^	0.0092 ± 0.0021 ^e^

## Data Availability

Sequencing data including gut microbiota 16S rDNA sequencing and metabolomics data have been deposited in Mendeley Data, V1, https://doi.org/10.17632/xb8y829cs9.1, accessed on 15 October 2025.

## Supplementary Materials

The following supporting information can be downloaded at: https://www.mdpi.com/article/10.3390/nu17213332/s1, Table S1. Preparation of Congo red experimental working liquid. Table S2. Preparation of Congo Red blank control working fluid. Table S3. PCR primer sequences for DNA detection. Table S4. Base size distribution in sequencing at 0 h and 48 h. Table S5. Effective sequencing data after quality control and chimera filtering. Table S6. Annotation information of key metabolites between the BLK and GFP groups. Figure S1. Comparison of α diversity of GFP after 48 h fermentation. (A–C) Rarefaction curves based on Chao1, Shannon, and Simpson indices; (D) Rank–abundance curve; (E–G) Diversity analysis using Chao1, Shannon, and Simpson indices. * *p* < 0.05; ** *p* < 0.01. Figure S2. Dominant microbial communities in each group after 48 h of fermentation. (A) Heatmap distribution of microbial abundance in each group; (B) Differences in microbial communities among the group; (C,D) Relative abundance of gut microbiota at the species level across the BLK, GFPs, and INL groups at 0 h and 48 h. Figure S3. Five key genera in the BLK group. Figure S4. Permutation test of PLS-DA scores among comparison groups. (A) GFP vs. BLK vs. INL; (B) GFP vs. BLK; (C) INL vs. BLK; (D) GFP vs. INL.
