# Supplementary material for: In Vitro Gastrointestinal Digestion of Grifola frondosa Polysaccharides and Their Enhancement of GABA Production via Gut Microbiota Modulation
Source: Nutrients. 2025 Oct 23;17(21):3332. doi: 10.3390/nu17213332 (PMC12610785; doi:10.3390/nu17213332)
Supplement: Supplementary file 1 [file nutrients-17-03332-s001.zip › Supplementary Data.pdf]

Supplementary Data

Table S1. Preparation of Congo red experimental working liquid.

NaOH (mol/L)	0	0.05	0.1	0.15	0.2	0.25	0.3	0.35	0.4	0.45	0.5
10 mg/mL GFP (mL)	0.9	0.9	0.9	0.9	0.9	0.9	0.9	0.9	0.9	0.9	0.9
300 μmol/L Congo red (mL)	0.5	0.5	0.5	0.5	0.5	0.5	0.5	0.5	0.5	0.5	0.5
Deionized water (mL)	1.6	1.45	1.3	1.15	1.0	0.85	0.7	0.55	0.4	0.25	0.1
1.0 mol/L NaOH (mL)	0	0.15	0.3	0.45	0.6	0.75	0.9	1.05	1.20	1.35	1.5

Table S2. Preparation of Congo Red blank control working fluid.

NaOH (mol/L)	0	0.05	0.1	0.15	0.2	0.25	0.3	0.35	0.4	0.45	0.5
300 μmol/L Congo red (mL)	0.5	0.5	0.5	0.5	0.5	0.5	0.5	0.5	0.5	0.5	0.5
Deionized water (mL)	2.5	2.35	2.2	2.05	1.9	1.75	1.6	1.45	1.3	1.15	1.0
1.0 mol/L NaOH (mL)	0	0.15	0.3	0.45	0.6	0.75	0.9	1.05	1.20	1.35	1.5

Table S3. PCR primer sequences for DNA detection.

Amplified fragments	Primer sequence
V3—V4	341F (5'-CCTACGGGNGGCWGCAG-3')<br>805R(5'-GACTACHVGGGTATCTAATCC-3')

Table S4. Base size distribution in sequencing at 0 h and 48 h.

	<200	200-300	300-400	400-500	>=500	Total
0h	5	149	259	1068801	0	1069214
48h	18	543	143	1141057	0	1141761

Table S5. Effective sequencing data after quality control and chimera filtering

Sample	Raw_Tags	Raw_Bases	Valid_Tags	Valid_Bases	Valid %	Q20 %	Q30 %	GC %
INL6	60982	30.49M	48010	20.15M	78.73	97.67	93.52	55.19
INL5	81162	40.58M	64380	26.93M	79.32	97.77	93.82	54.97
INL4	86662	43.33M	81532	33.58M	94.08	98.54	95.54	57.80
INL3	79443	39.72M	65075	27.41M	81.91	98.06	94.45	55.19
INL2	87996	44.00M	83079	34.19M	94.41	97.89	93.42	57.92
INL1	80664	40.33M	65772	27.69M	81.54	97.91	94.19	55.12
GFP6	75970	37.98M	59759	25.27M	78.66	98.01	94.35	54.45

GFP5	74102	37.05M	58422	24.70M	78.84	97.91	94.05	54.47
GFP4	80424	40.21M	63098	26.65M	78.46	97.86	93.99	54.35
GFP3	80803	40.40M	63761	26.96M	78.91	97.95	94.25	54.53
GFP2	78584	39.29M	60085	25.43M	76.46	98.02	94.37	54.36
GFP1	84488	42.24M	67549	28.35M	79.95	97.32	92.80	54.47
BLK6	83119	41.56M	60528	25.83M	72.82	97.29	92.06	54.91
BLK5	82157	41.08M	57978	24.73M	70.57	98.32	95.03	54.83
BLK4	86228	43.11M	62248	26.56M	72.19	98.34	95.11	54.85
BLK3	64510	32.26M	59836	25.52M	92.75	98.34	95.10	54.84
BLK2	83987	41.99M	59196	25.25M	70.48	98.42	95.30	54.81
BLK1	86254	43.13M	61453	26.22M	71.25	97.73	93.14	54.84

Table S6. Annotation information of key metabolites between the BLK and GFP groups

Name	gamma-Aminobutyric acid	L-4-Hydroxyglutamate semialdehyde	Adenine	Guanine	Indoxyl	L-Tryptophan	5-Hydroxy-L-tryptophan
m/z	102.06	148.06	136.06	152.06	132.04	205.10	221.09
RT	0.82	0.62	1.25	1.25	2.96	2.13	2.01
GFP1	89778946.92	106713548.53	75724890.22	29834816.33	1576723.17	294170288.85	10274065.63
GFP2	86252093.53	119679046.31	62663009.64	25828232.73	1602287.41	292315016.56	10512759.80
GFP3	87510473.91	118338931.73	62726607.98	28790203.83	1501204.65	297263051.33	12078685.06
GFP4	87077341.05	116063771.96	68845668.94	27460155.22	1736533.39	260615053.64	12001727.70
GFP5	80074257.43	115512122.08	92097217.79	33862118.15	1496655.63	259489523.09	18655811.14
GFP6	90887135.40	108057863.44	97871227.09	34465684.56	1781078.62	317538772.94	17627793.23
BLK1	33291520.64	34299917.03	141418581.72	1912971.69	4968089.67	23634933.15	5708477.21
BLK2	34160056.77	29653316.45	159492285.77	588075.01	4265803.71	47419299.43	5793590.58

BLK3	34892780.03	30248320.81	160780564.48	3490876.74	5141903.01	25755878.35	11023864.55
BLK4	37248383.53	32573227.21	147615401.35	1997076.85	5529813.38	16403771.10	5099891.48
BLK5	32022266.70	27319232.44	161399543.25	2185773.23	5501296.88	21872101.31	10025300.44
BLK6	35956823.46	30334645.34	174775012.91	3181095.03	2681805.89	112714696.17	9426386.14
FC	6.71	3.71	0.47	13.50	0.49	2.35	2.42
p value	0.00	0.00	0.00	0.00	0.02	0.02	0.02
VIP	1.58	1.88	1.63	2.66	1.37	2.33	1.05

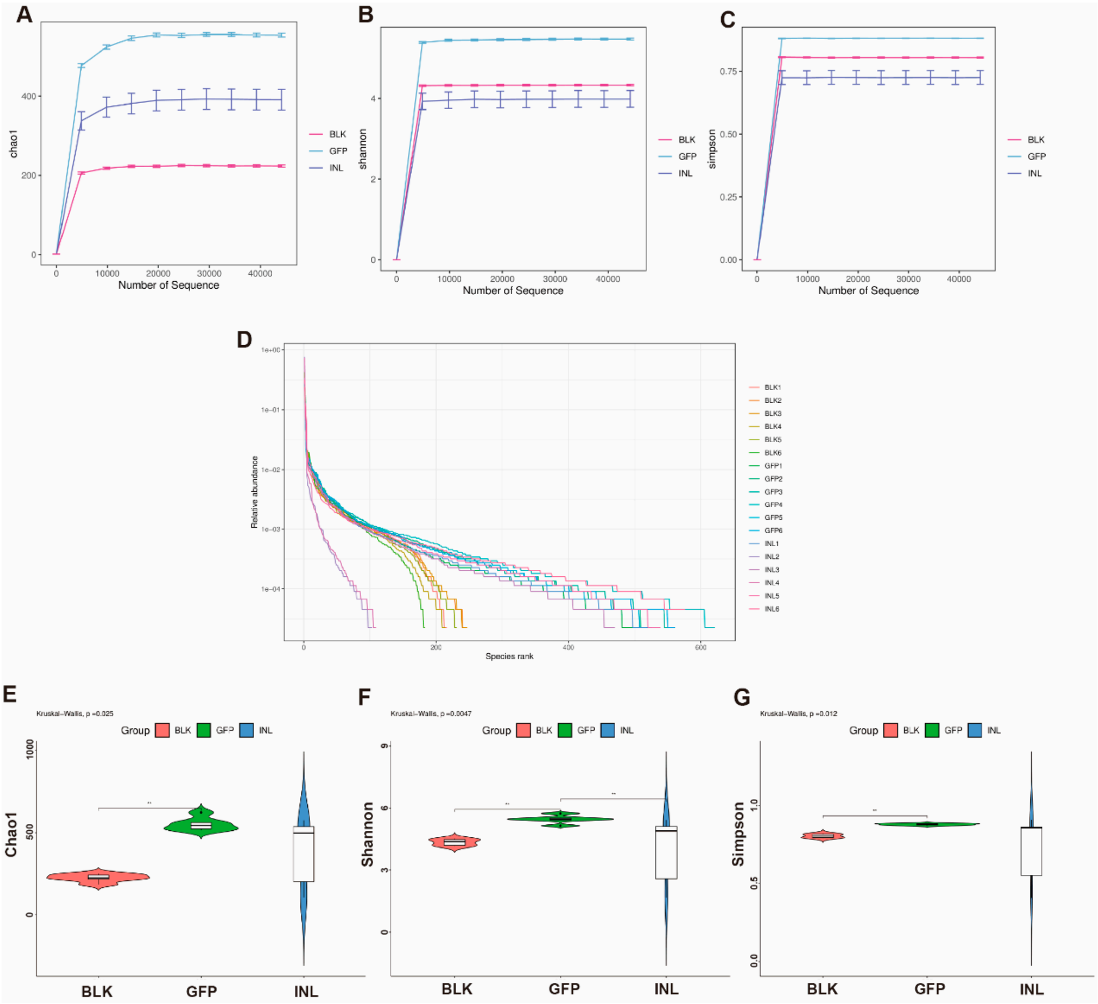

Figure S1. Comparison of α diversity of GFP after 48 h fermentation. (A–C) Rarefaction curves based on Chao1, Shannon, and Simpson indices; (D) Rank–abundance curve; (E–G) Diversity analysis using Chao1, Shannon, and Simpson indices. * $p < 0.05$; ** $p < 0.01$.

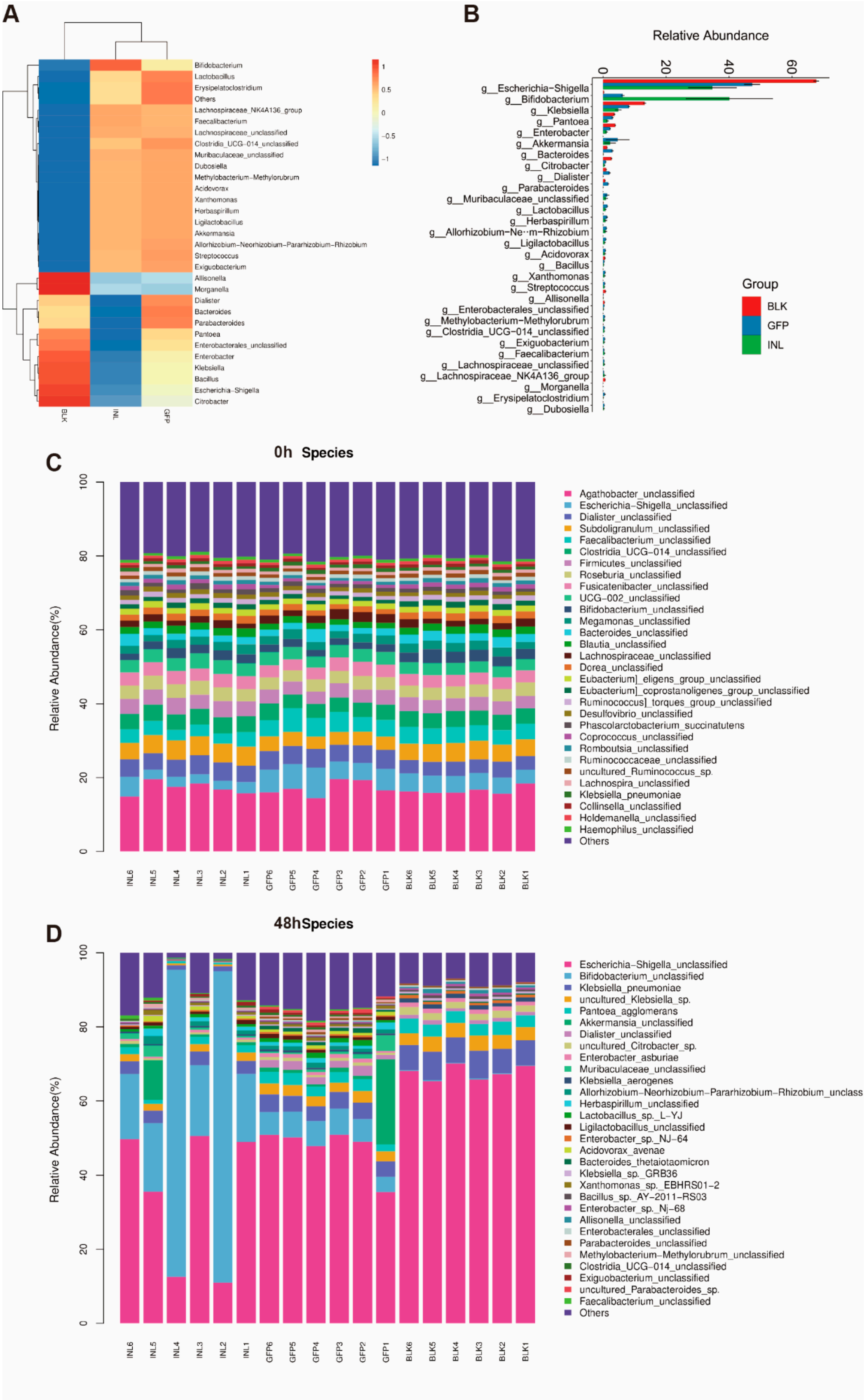

Figure S2. Dominant microbial communities in each group after 48 h of fermentation. (A) Heatmap distribution of microbial abundance in each group; (B) Differences in microbial

communities among the group; (C-D) Relative abundance of gut microbiota at the species level across the BLK, GFPs, and INL groups at 0 h and 48 h.

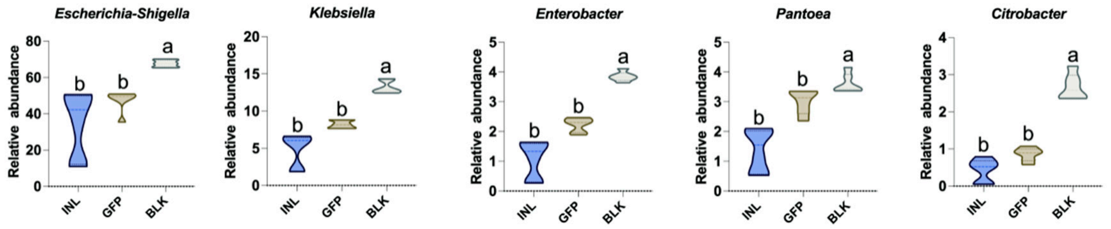

Figure S3. Five key genera in the BLK group.

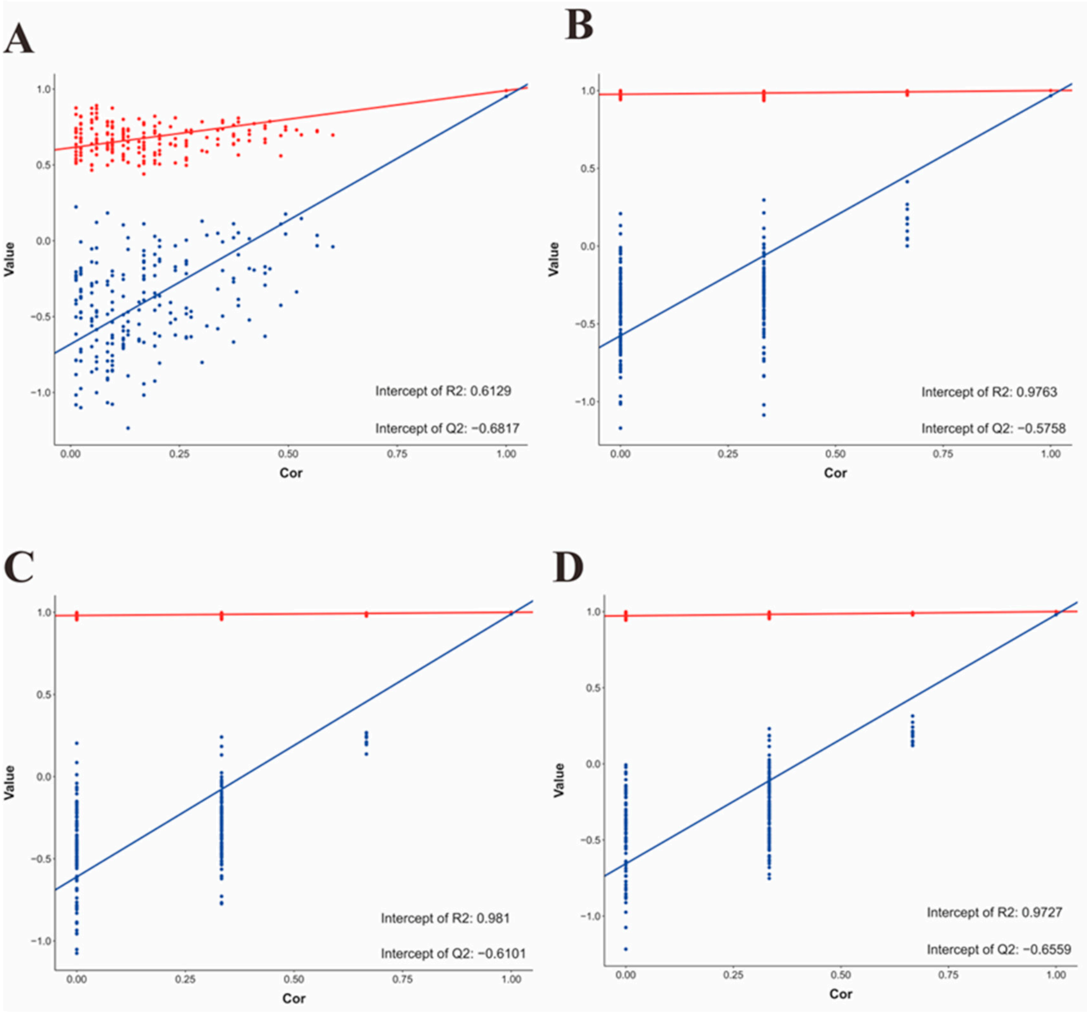

Figure S4. Permutation test of PLS-DA scores among comparison groups. (A) GFP vs. BLK vs. INL; (B) GFP vs. BLK; (C) INL vs. BLK; (D) GFP vs. INL.
